# FoPGDB: a pangenome database of *Fusarium oxysporum*, a cross-kingdom fungal pathogen

**DOI:** 10.1093/database/baae017

**Published:** 2024-03-27

**Authors:** Tan Meng, Hanqing Jiao, Yi Zhang, Yi Zhou, Shaoying Chen, Xinrui Wang, Bowen Yang, Jie Sun, Xin Geng, Dilay Hazal Ayhan, Li Guo

**Affiliations:** Peking University Institute of Advanced Agricultural Sciences, Shandong Laboratory of Advanced Agricultural Sciences in Weifang, Weifang, Shandong 261325, China; Department of Computer Science, The University of Hong Kong, Hong Kong 999077, China; Department of Computer Science, The University of Hong Kong, Hong Kong 999077, China; Peking University Institute of Advanced Agricultural Sciences, Shandong Laboratory of Advanced Agricultural Sciences in Weifang, Weifang, Shandong 261325, China; College of Information and Electrical Engineering, China Agricultural University, Haidian District, Beijing 100083, China; Peking University Institute of Advanced Agricultural Sciences, Shandong Laboratory of Advanced Agricultural Sciences in Weifang, Weifang, Shandong 261325, China; College of Information and Electrical Engineering, China Agricultural University, Haidian District, Beijing 100083, China; Peking University Institute of Advanced Agricultural Sciences, Shandong Laboratory of Advanced Agricultural Sciences in Weifang, Weifang, Shandong 261325, China; Peking University Institute of Advanced Agricultural Sciences, Shandong Laboratory of Advanced Agricultural Sciences in Weifang, Weifang, Shandong 261325, China; Peking University Institute of Advanced Agricultural Sciences, Shandong Laboratory of Advanced Agricultural Sciences in Weifang, Weifang, Shandong 261325, China; Peking University Institute of Advanced Agricultural Sciences, Shandong Laboratory of Advanced Agricultural Sciences in Weifang, Weifang, Shandong 261325, China; Peking University Institute of Advanced Agricultural Sciences, Shandong Laboratory of Advanced Agricultural Sciences in Weifang, Weifang, Shandong 261325, China; Peking University Institute of Advanced Agricultural Sciences, Shandong Laboratory of Advanced Agricultural Sciences in Weifang, Weifang, Shandong 261325, China; Peking University Institute of Advanced Agricultural Sciences, Shandong Laboratory of Advanced Agricultural Sciences in Weifang, Weifang, Shandong 261325, China

## Abstract

Pangenomes, capturing the genetic diversity of a species or genus, are essential to understanding the ecology, pathobiology and evolutionary mechanisms of fungi that cause infection in crops and humans. However, fungal pangenome databases remain unavailable. Here, we report the first fungal pangenome database, specifically for *Fusarium oxysporum* species complex (FOSC), a group of cross-kingdom pathogens causing devastating vascular wilt to over 100 plant species and life-threatening fusariosis to immunocompromised humans. The *F. oxysporum* Pangenome Database (FoPGDB) is a comprehensive resource integrating 35 high-quality FOSC genomes, coupled with robust analytical tools. FoPGDB allows for both gene-based and graph-based exploration of the *F. oxysporum* pangenome. It also curates a large repository of putative effector sequences, crucial for understanding the mechanisms of FOSC pathogenicity. With an assortment of functionalities including gene search, genomic variant exploration and tools for functional enrichment, FoPGDB provides a platform for in-depth investigations of the genetic diversity and adaptability of *F. oxysporum*. The modular and user-friendly interface ensures efficient data access and interpretation. FoPGDB promises to be a valuable resource for *F. oxysporum* research, contributing to our understanding of this pathogen’s pangenomic landscape and aiding in the development of novel disease management strategies.

**Database URL**: http://www.fopgdb.site

## Introduction

Fungal pathogens are eukaryotic microbes and major causal agents of devastating plant diseases threatening crop yields and food security. Many of them are also producers of mycotoxins poisoning agricultural produce and triggering genetic disorders such as cancers. *Fusarium oxysporum* species complex (FOSC) is a group of soil-borne filamentous fungal pathogens that cause devastating diseases in a wide range of plants, impacting global agricultural production (1–3). *F. oxysporum* infects the vascular system of host plants, leading to wilt, necrosis and ultimately plant death (3). FOSC has shown remarkable adaptability, with numerous formae speciales capable of infecting specific plant species or cultivars. As a result, it poses a significant threat to various economically important crops, including banana, tomato, cotton, melons, chickpea, crucifers and many others. It can also infect animals, causing fatal infection in immunocompromised humans (4, 5). The genomes of FOSC are highly variable due to the presence of various numbers of accessory chromosomes horizontally transferred among different FOSC strains, making them one of the most genetically diverse fungal pathogens. One of the main players in the plant–pathogen interface is the effectors, a group of small secreted proteins (2). *F. oxysporum* effectors are recruited during the infection course and play important roles in manipulating and evading the host’s immune system, ensuring the success of the fungal infection. The *F. oxysporum* accessory chromosomes are enriched with effectors and rich in repetitive elements primarily composed of transposable elements (TEs) whose active transposition leads to quick evolution of effectors and thus pathogenicity. Understanding the biology of the effectors not only provides insights into the molecular mechanisms of FOSC pathogenicity but also holds promise for the development of new strategies to control this devastating fungal pathogen.

Single-reference genome-based research provides a limited look into the organism’s adaptation. Therefore, genomic research has slowly shifted its paradigm from single genome to pangenomes which represent the genetic diversity of a species or genus, in order to overcome the reference bias (6). Given the quick evolution of *F. oxysporum* virulence, compartmentalized genome structure and importance of pangenomes, building a pangenome reference from high-quality *F. oxysporum* genome assemblies is essential to understanding the genetic diversity and evolution of pathogenicity for disease control. Many genome assemblies have been reported for *F. oxysporum* strains in recent years with 35 genomes with scaffold-level assemblies available in public domains such as National Center for Biotechnology Information (NCBI) GenBank and Joint Genome Institute (JGI). However, no graph pangenome reference has been available for *F. oxysporum* which has limited the integration and application of these genomic resources for pathobiological studies. Similar strategies have been employed for *F. oxysporum* as well (7, 8). The pangenomic approach is especially desirable for the compartmentalized genomes of FOSC (9) given their genetic diversity, host specificity and ability to quickly adapt to diverse environments. The pangenomes can be presented either as linear forms which contain the nonredundant sequences of all genomes or as a graph which utilizes graph data structure to store sequences and their relationships as nodes and edges, respectively. One tool that can help researchers to untangle the genome of FOSC is graph-based pangenomes (10). These powerful tools facilitate structural variation (SV) studies and can help us understand the genome plasticity of the *F. oxysporum* genome.

Here, we report a database for the gene-based and graph-based pangenome of the FOSC which the users can access and search the data and analysis and utilize various tools. The database contains 35 high-quality publicly available annotated genome sequences, putative effector sequences, core and dispensable gene categories, Kyoto Encyclopedia of Genes and Genomes (KEGG) and Gene Ontology (GO) term enrichment tools, genome browser, etc. We also report the first pangenome graph for FOSC that can be downloaded or browsed on the website.

## Materials and methods

### Data

To construct the FOSC pangenome, we collected 35 high-quality genomes of plant pathogenic and nonpathogenic strains as well as clinical isolates of *F. oxysporum*. The genomes were selected and downloaded from NCBI (9 genomes), JGI (24 genomes) or National Genomics Data Center(2 genomes) if assembled using at least one type of third-generation sequencing data such as PacBio circular consensus sequencing (CCS) or Oxford Nanopore sequencing reads and with genome annotations (Table 1) (11–21). The genome sizes range between 48.4 and 73.3 Mb, with a mean contig N50 of 3.6 Mb, while the numbers of genes range from 15 519 to 21 781.

**Table 1.** Statistics of the genome used to construct the FoPGDB

Genome	Genome size (Mb)	N50 (Mb)	Gene number	Reference
*F. oxysporum* f. sp. *albedinis* isolate 9	65.6	2.9	19 411	(18)
*F. oxysporum* f. sp. *basilici* Amherst-23	58.0	4.2	18 527	np
*F. oxysporum* f. sp. *basilici* Amherst-33	56.7	1.1	18 076	np
*F. oxysporum* f. sp. *basilici* Amherst-72	58.1	6.0	18 933	np
*F. oxysporum* f. sp. *cepae* FoC_Fus2_v1	53.4	4.1	18 852	(12)
*F. oxysporum* f. sp. *cubense* Foc1 60	48.6	4.7	15 865	(16)
*F. oxysporum* f. sp. *cubense* FocTR4 58	48.4	4.4	15 519	(16)
*F. oxysporum* f. sp. *lycopersici* 4287	55.6	4.1	17 932	np
*F. oxysporum* f. sp. *matthiolae* PHW726	57.2	0.8	17 996	(17)
*F. oxysporum* f. sp. *pisi* F105	71.5	0.8	19 358	np
*F. oxysporum* f. sp. *pisi* F109	67.1	1.0	19 272	np
*F. oxysporum* f. sp. *pisi* F23	73.3	0.7	20 063	np
*F. oxysporum* f. sp. *pisi* F79	67.0	0.9	19 180	np
*F. oxysporum* f. sp. *pisi* T415	55.8	3.6	18 001	np
*F. oxysporum* f. sp. *radicis-cucumerinum* Forc016	52.9	4.5	16 795	(14)
*F. oxysporum* f. sp. *vasinfectum* 15-2J	64.5	4.4	18 377	np
*F. oxysporum* f. sp. *vasinfectum* ME23	49.9	4.9	16 610	np
*F. oxysporum* f. sp. *vasinfectum* Tm2	61.1	4.7	17 844	np
*F. oxysporum* f. sp. *cubense* II5	49.4	4.5	16 048	(21)
*F. oxysporum* F10-03	56.6	4.6	17 114	np
*F. oxysporum* F10-04	57.9	5.8	18 482	np
*F. oxysporum* F10-08	56.0	4.3	17 958	np
*F. oxysporum* F10-10	53.0	3.9	16 942	np
*F. oxysporum* F2-01	56.6	3.6	17 966	np
*F. oxysporum* F2-02	55.4	5.3	17 736	np
*F. oxysporum* F2-04	56.6	4.5	17 876	np
*F. oxysporum* F2-06	55.3	4.8	17 672	np
*F. oxysporum* F2-07	54.0	4.7	17 461	np
*F. oxysporum* Fo47	50.4	4.5	17 426	(13)
*F. oxysporum* Fo5176	68.4	4.1	21 683	(20)
*F. oxysporum* MPI-CAGE-CH-0212	55.7	4.8	17 726	(15)
*F. oxysporum* MPI-SDFR-AT-0094	52.2	1.6	17 445	(15)
*F. oxysporum* MRL8996	50.1	1.7	16 631	(19)
*F. oxysporum* NRRL 26 365	48.5	0.7	16 047	np
*F. oxysporum* f. sp. *conglutinans* Cong1-1	72.2	4.9	21 781	(11)

np: unpublished.

### Gene and repeat annotations

KEGG and GO terms were annotated via eggnog-mapper (22, 23). In addition, the putative effector proteins in all genomes were annotated using SignalP version 5.0b (24), TargetP version 2.0 (25) and EffectorP version 3.0 (26). Briefly, the intersecting proteins between the secreted proteins detected by SignalP and TargetP, and the predicted effectors by EffectorP were annotated as putative effectors. In total, 20 728 putative effectors have been identified. The repeats were identified *de novo* by RepeatModeller (version 2.0.3) (27) using universal Repbase database and automatically curated by MCHelper (28) using default parameters. RepeatMasker (version 4.1.2-p1) (29) was used to annotate the repetitive elements in the genome using curated TE library.

### Gene-based pangenome

Orthofinder was used to cluster 628 715 protein sequences from 35 genomes into 29 569 gene families. The single-copy orthologs were used to generate a maximum-likelihood tree with *Fusarium graminearum* (GenBank accession: GCA_000240135.3) as outgroup. Clustered families were divided into five categories: core (present in all 35 genomes, 30.4% of the families), soft-core (present in exactly 34 genomes, 6.2%), dispensable (present in 3 to 33 genomes, 32.3%), peripheral (present in 2 or 3 genomes, 14.5%) and private (present in only 1 genome, 16.6%).

We performed KEGG and GO enrichment analyses of the genes in five categories, with distinct terms of significant enrichment. For example, while in the core category, conserved biological processes such as protein biosynthesis, ubiquitination, and DNA synthesis genes were enriched, the dispensable, peripheral, and private categories were enriched in diverse terms such as intracellular distribution of mitochondria, calcium ion import into vacuole, long-chain fatty acid metabolic process, or gamma-aminobutyric metabolic process. It must be noted that the annotated gene ratios for these categories were significantly lower than core and soft-core categories.

### Graph-based pangenome

The graph pangenome was constructed using two methods for different purposes. A SV graph was generated using Minigraph (version 0.19-r551) (29), with tomato pathogen Fol4287 as reference and otherwise default parameters. It contained 94 447 254 bases, 47 752 nodes and 60 911 edges and can be browsed using VPRG (https://github.com/codeatcg/VRPG). The main variation graph was generated using the Cactus-Minigraph pipeline (version 2.6.4) (30) with tomato pathogen Fol4287 as the reference and otherwise default parameters. It contains 694 513 706 bases, 18 390 096 nodes and 24 944 941 edges. The differences between the two versions reflect the complexity of the Cactus-Minigraph graph. vg deconstruct (version 1.43.0) (31) was used to call variants with all 35 genomes as reference. For Fol4287 as reference, a total of 1 575 210 single-nucleotide polymorphisms (SNPs) and small insertions and deletions (INDELs) and 17 677 SVs were detected. The variants can be browsed using JBrowse (see later).

### Genome alignments

Genome alignments were carried out using the Minimap2 (version 2.24) (32), which facilitated the alignment of the *F. oxysporum* f. sp. *lycopersici* 4287 genome with those of the other 34 genomes in our dataset. To provide a comprehensive overview of the alignment results, we employed the D-GENIES visualization tool (33). These alignments can be explored in details within the ‘Align’ under ‘Tools’ module.

### Database implementation

**Figure 1.**
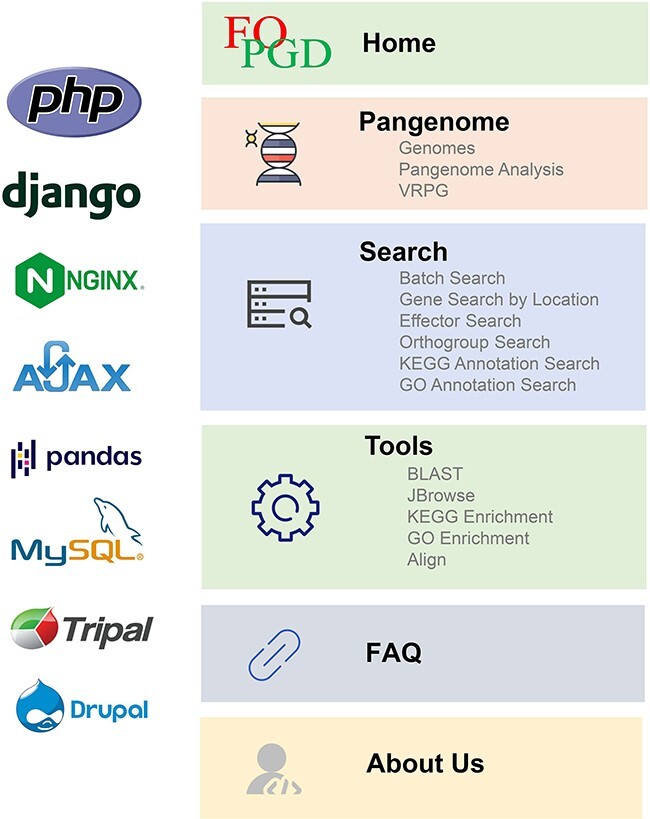
The framework, core system and programming language of FOPGDB.

The FoPGD, a robust pangenomic analysis platform for *F. oxysporum*, leveraged a range of technologies and frameworks for its construction and operation (Figure 1). For the construction of the Home and About Us pages and the implementation of BLAST and JBrowse in the Tools module, *F. oxysporum* Pangenome Database (FoPGDB) utilized Tripal (http://tripal.info/), a toolkit specifically designed for constructing online genomic and genetic databases. The web pages of FoPGDB were developed using HyperText Markup Language (HTML), Cascading Style Sheets (CSS) and JavaScript and administrated via Drupal, a PHP (Hypertext Preprocessor)-based website management system. The biological data were imported into the database utilizing the Generic Model Organism Database Project/Chado (34–36) schema, a widely used database schema for biological information. For the Search module, with the exception of the orthogroup search, the platform employed the Nginx (https://nginx.org/) web server, in conjunction with PHP, HTML5 and JavaScript as programming languages. For the organization, storage and management of data, FoPGDB deployed MySQL (https://www.mysql.com). Additionally, the AJAX asynchronous loading scheme was incorporated to enhance data loading efficiency and streamline function implementation. The remaining sections, including the pangenome, orthogroup search page and KEGG and GO enrichment tools, utilized Django (https://www.djangoproject.com), a high-level Python web framework. These pages incorporated HTML, CSS and JavaScript for front-end design. Data storage and manipulation were facilitated by pandas (https://pandas.pydata.org), an open-source data analysis and manipulation library renowned for its speed, flexibility and ease of use. Moreover, ClusterProfiler (37), an R package for the statistical analysis and visualization of functional profiles, and VRPG, an interactive web viewer for reference pangenome graphs, were implemented.

## Results

The FoPGDB website was organized into five main modules: the ‘Home’ module which contains general information as well as shortcuts to ‘Search’ and ‘Tools’ modules, ‘Pangenome’ module where the data and the analysis are hosted, ‘Search’ and ‘Tools’ modules which contain tools related to gene searches and alignments, genome browsers and genome alignments, ‘Frequently Asked Questions (FAQ)’ module, and finally the ‘About Us’ module where more information about the website is provided as well as contact information (Figure 1). In addition to the main modules, ‘External Links’ contains quick access links to third-party tools.

### Home, FAQ, and About Us

**Figure 2.**
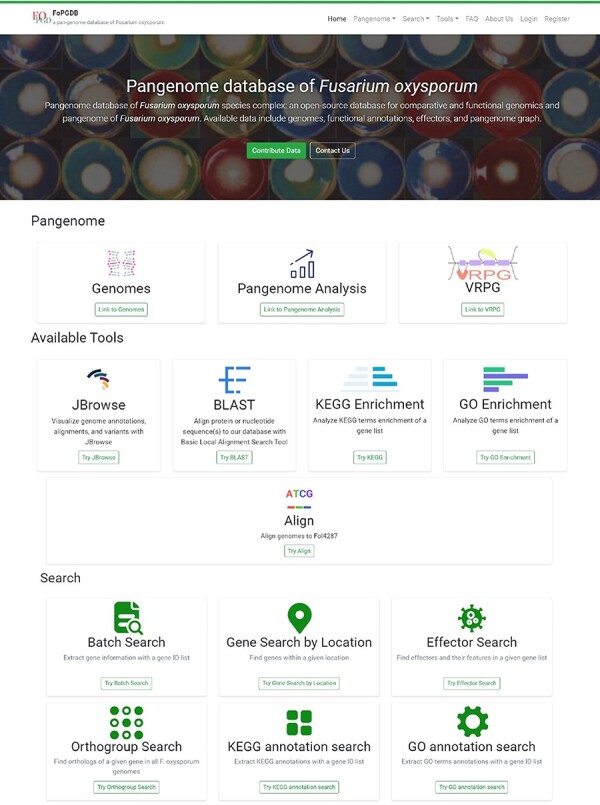
The homepage of FoPGDB.

The ‘Home’ page contains a short description of the database and quick access links to the ‘Pangenome’, ‘Search’ and ‘Tools’ modules (Figure 2). The background image is an artistic rendering of different FOSC strains in various media plates. In ‘FAQ’ module, we answer possible questions about the database and give a quick overview of the Tools. The ‘About Us’ module has more information about the database and contact information.

### Pangenome

The framework of the current study encompasses three principal components within the Pangenome module: ‘Genomes’, ‘Pangenome Analysis’ and ‘VRPG’.

#### Genomes

The ‘Genomes’ module delivers an exhaustive table depicting the genomes deployed for the study, accompanied by assembly statistics, references and immediate access to NCBI, National Genomics Data Center or JGI for the procurement of relevant data. Moreover, an integrated search function situated at the upper-right corner of the table facilitates effective data extraction from the entire dataset. To enhance user convenience, the table is equipped with a header-based sorting mechanism, thereby expediting the search and identification process for desired information.

#### Pangenome Analysis

**Figure 3.**
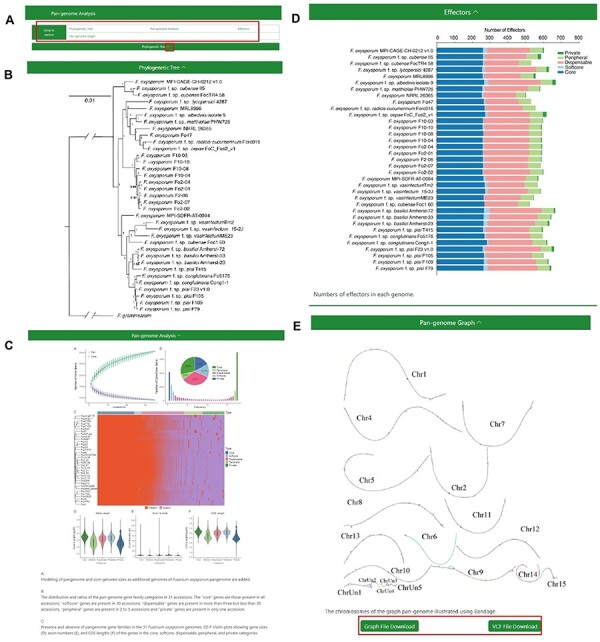
The Pangenome Analysis section under the Pangenome module. (A) Quick access links. (B) Maximum-likelihood tree using single-copy orthologous protein sequences in 35 *Fusarium oxysporum* genomes. *Fusarium graminearum* was used as an outgroup. (C) (Top left) Modeling of pangenome and core-genome sizes as additional genomes of *F. oxysporum* pangenome are added. (Top right) The distribution and ratios of the pangenome gene family categories in 35 accessions. The ‘core’ genes are those present in all accessions, ‘softcore’ genes are present in 34 accessions, ‘dispensable’ genes are present in more than 3 but less than 34 accessions, ‘peripheral’ genes are present in 2 to 3 accessions and ‘private’ genes are present in only 1 accession. (Center) Presence and absence of pangenome gene families in the 35 *F. oxysporum* genomes. (Bottom) Violin plots showing gene sizes (left), exon numbers (middle) and CDS lengths (right) of the genes in the core, soft-core, dispensable, peripheral and private categories. (D) Numbers of effectors in each genome. (E) Visualization of the chromosomes in the graph genome with Bandage.

The ‘Pangenome Analysis’ subdivision incorporates a ‘jump to section’ navigation bar, tactically placed beneath the primary menu (Figure 3A). This assists users in swiftly accessing specific sections of interest. The navigation bar is partitioned into four modules: ‘Phylogenetic Tree’, ‘Pan-genome Analysis’, ‘Effectors’ and ‘Pangenome Graph’ (Figure 3B–E) The initial three modules provide insights into the phylogenetic tree of the 35 FOSC genomes, particulars of the pangenome analysis and the distribution of effectors, respectively. Within the ‘Pangenome Graph’ section, users can visualize an overview of the pangenome graph and have the option to download the Graphical Fragment Assembly file and the Variant Call Format file, with Fol4287 serving as the reference genome. A concealment option, integrated into the title bar of each module, gives users enhanced content control.

#### VRPG

**Figure 4.**
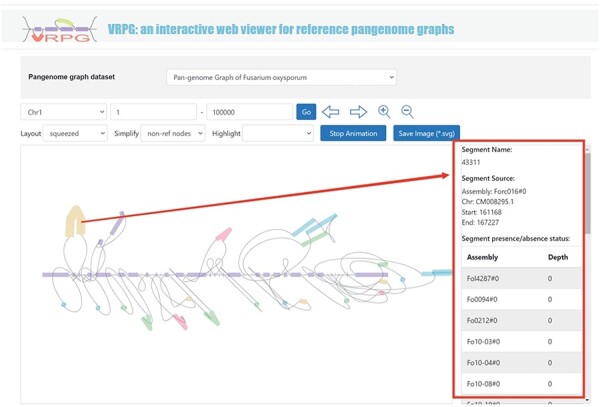
VRPG section under the Pangenome module shows a section in the graph pangenome.

The ‘Pangenome’ module further introduces a specialized subdivision named ‘VRPG’ (Figure 4). This provides an interactive and intuitive platform for visualizing the pangenome graph of *F. oxysporum*. The platform enables users to select specific regions of interest within the pangenome graph and refine image through easy-to-use input boxes and buttons. For improved visualization, a selection box allows for the highlighting of the genome used in constructing the pangenome graph. Customization features include the layout and simplification style of the visualized pangenome graph, thus offering customized visualizations. A unique function within this section allows users to click on small sequences within the graph and retrieve information about their positions and potential instances of these sequences in other genomes used to create the pangenome graph. An option to save the pangenome graph as an Scalable Vector Graphics image has been integrated.

### Search

The FoPGDB provides a sophisticated ‘Search’ section to facilitate the exploration of genes via gene IDs or specific genomic coordinates. This section comprises six robust search tools: ‘Batch Search’, ‘Gene Search By Location’, ‘Effector Search’, ‘Orthogroup Search’, ‘KEGG Annotation Search’ and ‘GO Annotation Search’ (Figure 6).

#### Batch Search and Effector Search

**Figure 5.**
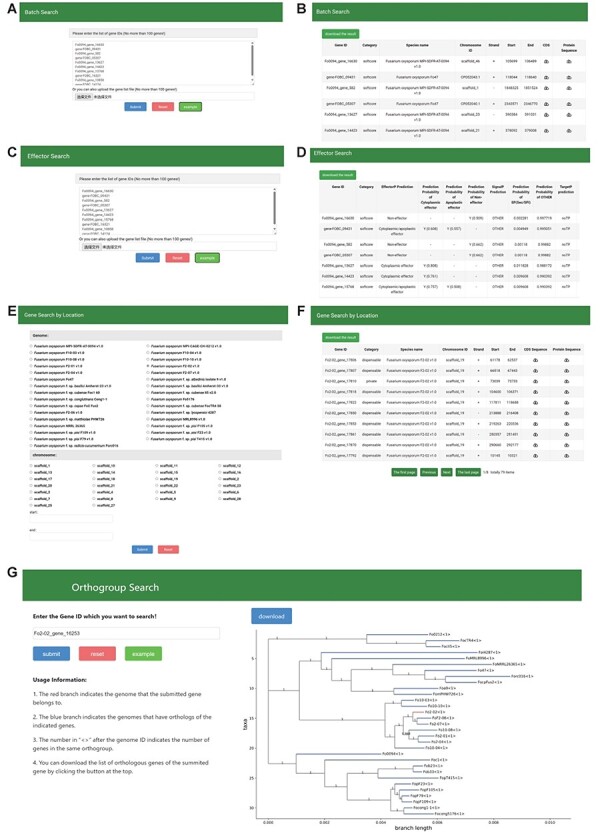
The Search module sections. (A, B) Search page and result page of Batch Search, (C, D) Search page and result page of effector search, (E, F) Search page and result page of Gene Search By Location, (G) Search page and result page of Orthogroup search.

The ‘Batch Search’ tool offers an efficient method for the retrieval of essential gene-related information, including gene categories, genomic locations, protein sequences and coding DNA sequences (CDS) (Figure 5A and B). Notably, the CDS information can be instrumental in designing PCR primers. Users can input gene IDs directly into a designated field or upload a file containing multiple gene IDs to use the ‘Batch Search’ feature. Similarly, the ‘Effector Search’ functionality also operates via the direct input of gene IDs or by uploading a file containing multiple gene IDs (Figure 5C and D). It allows users to access the effector prediction results associated with the provided gene IDs. These predictions are formulated based on algorithms such as SignalP, TargetP and EffectorP.

#### Gene Search By Location

The ‘Gene Search By Location’ function is designed to streamline the gene identification process within a specified genomic region (Figure 5E and F). Users can determine the genome and chromosome of interest from a selection box and enter the start and end positions in a separate input box, thus narrowing the genomic region for the search.

#### Orthogroup Search

The ‘Orthogroup Search’ offers a resource for users to examine orthologous genes across species (Figure 5G). Researchers may input a gene ID into the left-hand input box to visualize the distribution of orthologous genes within a phylogenetic tree. The database also allows the download of all gene information within an orthogroup in CSV format.

#### KEGG Annotation Search and GO Annotation Search

The ‘KEGG Annotation Search’ and ‘GO Annotation Search’ tools allow users to explore the KEGG pathway and GO term annotations for selected genes, respectively. These tools can enhance the understanding of gene function by providing direct access to KEGG and GO data upon gene ID entry. To optimize user interactions, sample gene lists are provided for the ‘Search’ subsections that require gene ID input. Users can populate the input box with a sample gene list by clicking the ‘example’ button. Further enhancing functionality, the database enables the download of search results in CSV format, providing a practical solution for data analysis and archiving.

### Tools

The ‘Tools’ module represents a pivotal component of our pangenome database, providing users with an array of powerful and user-friendly functionalities. This section is divided into five distinct and specialized subsections, namely: ‘BLAST’, ‘JBrowse’, ‘KEGG Enrichment’, ‘GO Enrichment’ and ‘Align’.

#### BLAST

**Figure 6.**
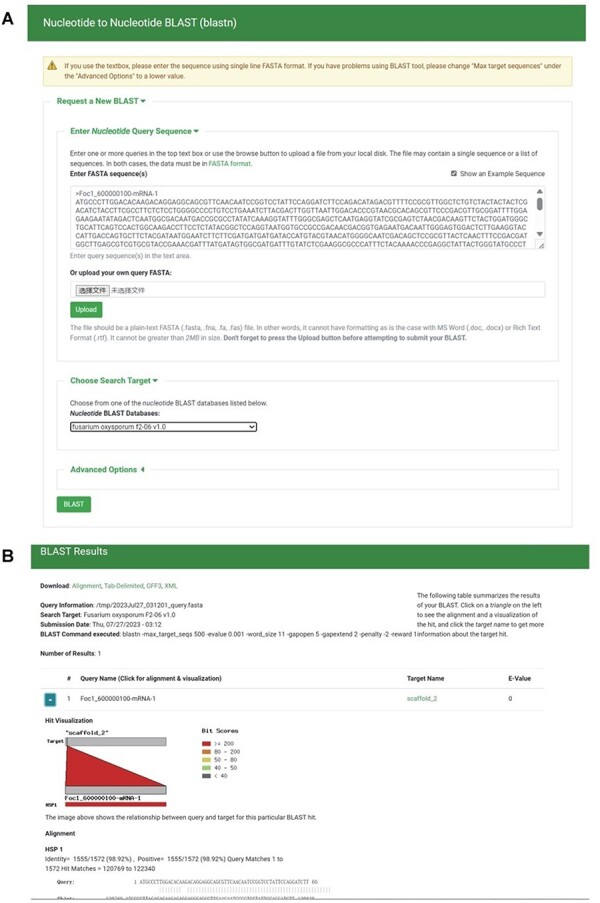
BLAST section under the Tools module. (A) BLAST search page. (B) BLAST result page.

To create local BLAST databases from the genome sequences, transcript sequences, protein sequences and TE sequences of the 35 *F. oxysporum* genomes, we utilized NCBI BLAST version 2.10.1+ (38). Employing the ‘tripal_blast’ module (https://github.com/tripal/tripal_blast), we developed an intuitive interface for BLAST searches (Figure 6A). The BLAST search results are presented as an expandable summary table, with each hit listed as a row, providing essential information such as query sequence ID, subject sequence ID and e-value (Figure 6B). For detailed alignment information, including hit visualization and high-scoring pairs between query and subject sequences, users can easily unfold the rows. Moreover, users have the option to download the BLAST search results in various formats, including BLAST pairwise format, BLAST tabular format, GFF3 and BLAST XML format.

#### JBrowse

**Figure 7.**
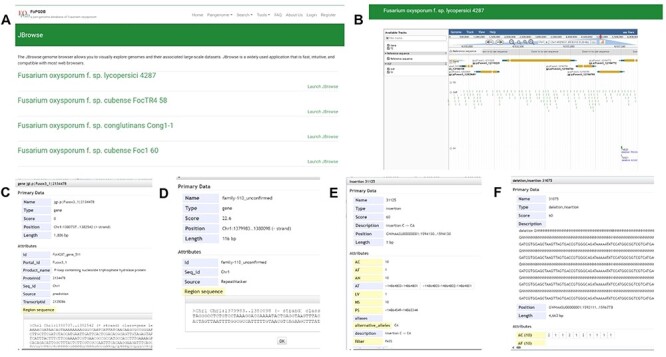
JBrowse section under the Tools module. (A) Page of genome browser index, (B) available tracks for different types of genomic features, (C) a window showing detailed information of the target gene model, (D) TE information, (E) SNPs and INDELs information for 35 *Fusarium oxysporum* accessions, (F) SVs’ information for 35 *F. oxysporum* accessions.

Incorporating JBrowse, a versatile genome browser supporting interactive access and visualization of various genomic features (39), we have crafted a custom JBrowse for each of the 35 genomes (Figure 7). Within each genome’s JBrowse, five tracks are offered, including genome sequences, gene models, repeats, SNPs and INDELs and SVs (Figure 7A and B). The genome structure, gene models and repeats provide insights into chromosomal locations, gene structures and sequences. As for the SNP&INDEL and SV track, derived from the pangenome graph using each genome as a reference with vg deconstruct, users can effortlessly explore SNPs, INDELs and SVs for the reference sequence in comparison with the pangenome graph. By clicking on specific variants within the ‘SNP&INDEL’ track and ‘SV’ track (Figure 7C–E), users can access information regarding chromosome locations, variation types and frequencies among the 35 genomes. For example, we investigated the methylation-related SNPs, INDELs and SVs located in the gene Fol4287_gene_849, allowing us to view gene position, length and sequence by clicking on the gene model (Figure 7C).

#### KEGG enrichment and GO enrichment

**Figure 8.**
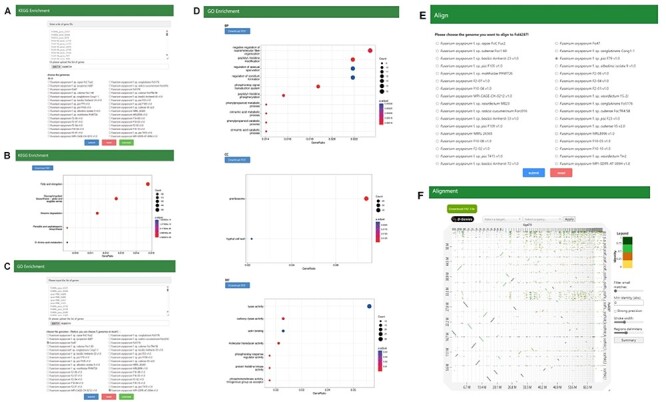
(A, B) Search page and result page of KEGG enrichment, (C, D) Search page and result page of GO Enrichment, (E, F) Search page and result page of Alignment.

Functional enrichment analysis represents a potent method for extracting meaningful biological insights from gene data. To assist users in capturing essential biological information related to genes, we have implemented KEGG and GO enrichment analysis tools based on the functional annotations described earlier and the clusterProfiler R package (Figure 8A–D). Users can easily input a list of genes of interest or upload a file containing gene lists to perform enrichment analyses. The results offer significantly enriched functional categories, providing valuable insights into the potential biological processes associated with the selected genes.

#### Genome Alignment Tool

Pairwise genome alignments can be visualized under Align section for the genomes in FoPGDB. Users can select the genome to align with the reference genome, Fol4287, and view and interact with the dot plot generated by D-Genies (33). The alignments can also be downloaded as pairwise mapping format file (Figure 8E and F).

## Discussion

The FoPGDB has been developed as a robust and comprehensive resource for the fungal research community, particularly those interested in the study of the FOSC. FoPGDB incorporated 35 high-quality annotated genomes of FOSC, a vast repository of putative effector sequences, and a multitude of tools to facilitate gene-based and graph-based exploration of the FOSC pangenome. Using FoPGDB, one can align their gene of interest to BLAST databases, search the hit genes in the pangenome database using batch search, determine if they are effectors using effector search, browse the genomic region and search for variations using JBrowse and interactively inspect the genomic region using VRPG. The efficient search module and advanced toolsets allow for the detailed investigation of the genetic diversity, adaptability and pathogenicity of *F. oxysporum*.

As we look ahead, we plan to continually update and expand FoPGDB to enhance its utility and relevance for *F. oxysporum* research. Future additions will aim to incorporate more genomes as they become available, update the database with new effector sequences and improve the analytical tools based on user feedback and technological advancements. This will enable researchers to delve deeper into the pangenomic landscape of this pathogen, thereby facilitating the development of innovative disease management strategies. We believe that FoPGDB holds immense potential to propel the field of *Fusarium* research forward, by aiding in the exploration of genomic diversity and shedding light on the underlying mechanisms of pathogenicity.

## Data Availability

Data are available at FoPGDB (http://www.fopgdb.site) online.
